# Impact of Viewing *vs.* Not Viewing a Real Forest on Physiological and Psychological Responses in the Same Setting

**DOI:** 10.3390/ijerph111010883

**Published:** 2014-10-20

**Authors:** Masahiro Horiuchi, Junko Endo, Norimasa Takayama, Kazutaka Murase, Norio Nishiyama, Haruo Saito, Akio Fujiwara

**Affiliations:** 1 Division of Human Environmental Science, Mt. Fuji Research Institute, 5597-1, Kami-Yoshida, Fuji-Yoshida City, Yamanashi 4030005, Japan; E-Mail: jendo@mfri.pref.yamanashi.jp; 2 Department of Forest Management, Forestry and Forest Products Research Institute, 1 Matsuno-sato, Tsukuba City, Ibaraki 305-8687, Japan; E-Mail: hanri@ffpri.affrc.go.jp; 3 Fuji Iyashinomoroi Woodland Study Center, The University of Tokyo, Yamanaka 341-2, Yamanakako Village, Minami-tsuru, Yamanashi 4010501, Japan; E-Mails: murase@uf.a.u-tokyo.ac.jp (K.M.); nishi@uf.a.u-tokyo.ac.jp (N.N.); haruo_s@uf.a.u-tokyo.ac.jp (H.S.); akio@uf.a.u-tokyo.ac.jp (A.F.)

**Keywords:** blood pressure, cerebral oxygenation, mood states, visual stimulation, environmental planning

## Abstract

We investigated the impact of viewing* versus* not viewing a real forest on human subjects’ physiological and psychological responses in the same setting. Fifteen healthy volunteers (11 males, four females, mean age 36 years) participated. Each participant was asked to view a forest while seated in a comfortable chair for 15 min (Forest condition)* vs.* sitting the same length of time with a curtain obscuring the forest view (Enclosed condition). Both conditions significantly decreased blood pressure (BP) variables,* i.e.*, systolic BP, diastolic BP, and mean arterial pressure between pre and post experimental stimuli, but these reductions showed no difference between conditions. Interestingly, the Forest viewing reduced cerebral oxygenated hemoglobin (HbO_2_) assessed by near-infrared spectroscopy (NIRS) and improved the subjects’ Profile of Mood States (POMS) scores, whereas the Enclosed condition increased the HbO_2_ and did not affect the POMS scores. There were no significant differences in saliva amylase or heart rate variability (HRV) between the two conditions. Collectively, these results suggest that viewing a real forest may have a positive effect on cerebral activity and psychological responses. However, both viewing and not viewing the forest had similar effects on cardiovascular responses such as BP variables and HRV.

## 1. Introduction

Epidemiological studies have demonstrated that a green environment is associated with improvements in people’s self-esteem and mental health [1,2] and increased longevity of aged people living in urban areas [3]. An immersive forest experience known as “*shinrin-yoku*” in Japanese and sometimes called “forest bathing,” has received widespread attention as a novel form of therapy. Numerous studies examining the effects of forest environments on psychological states such as emotions and moods [4,5,6,7,8] and on physiological factors such as cerebral activity [5], heart rate variability (HRV) [6,7,8], pulse rate and blood pressure (BP) [6,7,8], and salivary amylase (sAMY) activity [9] have shown positive outcomes. However, a majority of the previous field studies compared forest and urban environments with respect to their physiological and psychological benefits for inhabitants [4,5,6,7,8,9]. It has been difficult to identify which factors of forest environments can have positive effects for human health.

A landmark study proposed that viewing nature *per se* could produce positive health effects in hospitalized individuals [10], which indicates that visual stimuli may strongly influence human health. Indeed, Park* et al.* (2007) [5] reported that total hemoglobin (Hb) at the frontal lobe assessed by near-infrared spectroscopy (NIRS) decreased significantly more after forest viewing than after urban viewing, suggesting that forest viewing has a relaxing effect. In addition, screen image studies have shown that pleasant feelings decrease cerebral oxygenated hemoglobin (HbO_2_), and unpleasant feelings increase it [11]. These results indicate that a visual stimulus such as a forest or a screen image may cause a relaxing effect.

Although highly controlled laboratory studies may help clarify the mechanisms underlying the positive effects of a forest environment on physiological and psychological responses, field studies are required to more precisely determine the actual health-related benefits of exposure to forest environments. This is because the results of field studies may be more generalizable than laboratory studies [12]. In addition, a comparative study within the same forest may generate novel information compared to a comparative study between natural and artificial environments. Such a comparative study may also provide information that is useful for environmental planning, e.g., for the creation of effective forest environments in urban areas. 

With this background in mind, we investigated the impact of viewing a real forest on human subjects’ physiological and psychological responses. We hypothesized that viewing a real forest would have greater positive effects on physiological and psychological states, in particular, cerebral HbO_2_ and total Hb assessed by NIRS, than not viewing a forest. To test this, we compared NIRS signal responses as well as blood pressure (BP), heart rate variability (HRV), and sAMY responses (which have been evaluated in previous studies) in the same forest setting.

## 2. Methods

### 2.1. Subjects

The subjects were 15 healthy volunteers (11 men and four women) with a mean ± SD age of 36 ± 8 years. All had no history of cardiovascular disease or mental illness. None had been taking any medications which could affect physiological or psychological responses related to BP changes, stress markers, and mood states. After receiving a detailed description and explanation of the study procedures and the possible risks and benefits of participation, each subject signed an informed consent form. They were asked to abstain from consuming caffeinated beverages for 12 h and to not engage in strenuous exercise or consume alcohol for a minimum of 24 h before the experiment. We conducted statistical power analysis using a “G*power 3.1.9.2” free software. As a result, actual power (1-β error probability) was 0.575 using a “statistical test of repeated measures, within-between interaction” and using a power analysis type of “Post hoc: Compute achieved power”, when effect size is 0.25, α error of probability is 0.05. However, several previous studies have demonstrated that the sample size of 12–15 subjects was enough to derive significance conclusions [5,7,8]. All procedures used in the present study were approved by the ethical committee of the Mt. Fuji Research Institute and were performed in accord with the guidelines of the Declaration of Helsinki.

### 2.2. Experimental Stimuli

Each subject underwent both the Forest and Enclosed conditions in random order with a 30-min interval between conditions: viewing a real forest, and not viewing the real forest. We selected one forest site, located near the Village of Yamanaka Lake in Yamanashi, Japan as the experimental setting. The forest’s vegetation was 66% Japanese larch, 10% giant dogwood, 7% Japanese red pine, 6% fire tree, and 11% other vegetation depending on the chest height of the cross-sectional area.

**Figure 1:**
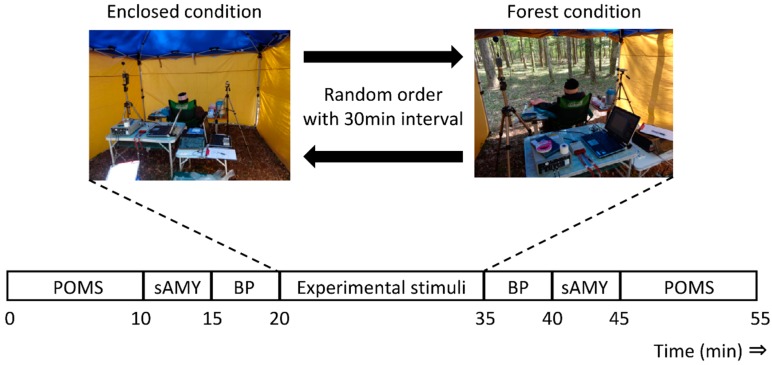
Experimental set-up of the Enclosed and Forest conditions. POMS, Profile of Mood States; sAMY, saliva amylase; BP, blood pressure.

As shown in Figure 1, cloth sheets were hung on both sides of the seated subject for the duration of the study, and another sheet of cloth was hung about 2 m in front of the subject as a curtain that obscured the subject’s view of the forest. By opening and closing the curtain in front of the subject, the viewing of the forest (Forest condition) and the non-viewing of the forest (Enclosed condition) were achieved (Figure 1).

### 2.3. Procedure

The physiological and psychological responses of each subject were measured for both the Forest and Enclosed conditions. After the attachment of all measuring devices, each subject underwent the viewing and non-viewing condition study. The subject’s BP variables, saliva amylase (sAMY), and Profile of Mood States (POMS) scores were evaluated before and after both conditions. Heart rate (HR) and near-infrared spectroscopy (NIRS) signals were measured throughout each 15-min experimental stimulus. For the pre-condition measurements, the subject sat on a comfortable chair in an upright position and completed the POMS questionnaire. His or her saliva was then collected and BP was measured with the subject in this position. The subject was instructed to view the landscape in front of him/her that either included or did not include the forest view, while sitting on the chair for 15 min. After the 15-min experimental stimulus, post-measurements were taken;* i.e.*, BP measurement, saliva collection, and the POMS questionnaire.

### 2.4. Measurements

We also measured the environmental conditions (*i.e.*, temperature and relative humidity) using a portable amenity meter (AM-101, Kyoto Electronic Mfg., Kyoto, Japan). The illumination intensity was measured using an illuminometer (T-10, Konica Minolta, Tokyo). These parameters were measured throughout the 15-min experimental stimuli every 5 min for each subject.

Systolic and diastolic blood pressure (SBP and DBP) values were measured from the left upper arm of each subject using a digital blood pressure monitor (HEM-7011, Omron, Tokyo) with the subject in a seated position on a comfortable chair, by an oscillometric method. BP was measured twice at each period,* i.e.*, pre-and post-measurement for both the Forest and Enclosed conditions. Average BP values of two measurements were taken as BP values.

HR was measured using a Check-My-Heart handheld HRV device (Daily Care BioMedical, Chungli, Taiwan) [13,14,15]. The electrodes of the handheld HRV device were attached to the subject’s lower left rib and right clavicle using a lead electrocardiogram (ECG) signal. The recordings were transferred to a computer, and the data for each 5-min ECG signal were analyzed automatically by HRV analysis software (Daily Care BioMedical). The 5-min HRV data were used in the statistical analysis. HR was recorded throughout the experiment. In the frequency domain, the extent of very-low-frequency oscillations (VLF: 0.0033–0.04 Hz), low-frequency oscillations (LF: 0.04–0.15 Hz), and high-frequency oscillations (HF: 0.15–0.4 Hz) were quantified using the fast Fourier transformation [16]. Thereafter, HF power was defined as an indicator of parasympathetic nerve activity, and the ratio of LF and HF values (*i.e.*, LF/HF) was used as an indicator of sympathetic nerve activity.

We measured the cerebral oxygenation and deoxygenation profiles of each subject’s left frontal lobe using NIRS (BOM, L1-TRW, Omegawave, Tokyo). This instrument uses three laser diodes (780, 810, and 830 nm) and calculates the relative tissue levels of oxygenated hemoglobin (HbO_2_) and deoxygenated hemoglobin (HHb) according to the modified Beer-Lambert law [17]. Total Hb was defined as the sum of HbO_2_ and HHb. Optodes were placed on the subject’s left frontal lobe, and a black cloth was wrapped around the probe holder to shield it from ambient light. The subject’s entire head was wrapped with an elastic bandage. The probe holder contained one light source probe, and two detectors were placed 2 cm (detector 1) and 4 cm (detector 2) away from the source. The Hb concentrations received by detector 1 were then subtracted from those received by detector 2. This procedure allowed us to minimize the influence of skin blood flow [18,19]. NIRS signals were measured at 1-s intervals throughout the experiment. In this regard, decreases in HbO_2_ and/or total Hb indicate a relaxing effect [5,11] as these reductions are associated with a decrease in cerebral blood flow (CBF) [20]; a pleasant feeling causes a decrease in CBF [21].

The sAMY concentration, which has been established as a marker of stress [22], was measured using a hand-held sAMY monitor (CM-2.1, NIPRO Co. Ltd, Osaka, Japan). Since it has been reported that increased sympathetic nerve activity is a major stimulator of amylase secretion, increases in sAMY indicate an increase of stress [9,22]. This hand-held monitor consists of a disposable test strip and an optical analyzer containing an automatic saliva transcription device. A volume of 20–30 mL of saliva was collected by the collecting paper placed under the subject’s tongue. The time allocated to saliva collection was 30 s for each subject, and it took 30 s to transfer and analyze the saliva using the hand-held monitor.

The Profile of Mood States (POMS) is a well-established, factor-based and analytically derived measure of psychological distress. Its reliability and validity have been well documented [23]. The POMS measures six mood states: Tension and Anxiety (*T-A*), Depression (*D*), Anger and Hostility (*A–H*), Vigor (*V*), Fatigue (*F*) and Confusion (*C*). We used the short Japanese version of the POMS (covering 30 items) [24] and its raw-score for our statistical analysis

### 2.5. Data and Statistical Analysis

The environmental condition parameters were calculated as average values for the 15 min during the experimental stimuli. Mean arterial pressure (MAP) was calculated as [(2 × diastolic pressure) + systolic pressure]/3 [17]. The HRV values for the first 5 min and the last 5 min of each condition were compared. NIRS signals were averaged for each 1-min measurement, and the first-minute values were defined as baseline values of zero. Thus, to compare the NIRS signals across the subjects, we used these signals to represent the changing rates from the baseline values given that NIRS signals can only be understood as relative values.

The data are expressed as the mean values ± the standard error of the mean (SEM). A paired t-test was conducted for comparisons between the viewing (Forest) and non-viewing (Enclosed) conditions. A two-way repeated measures analysis of variance (ANOVA) was used for comparisons of physiological and psychological responses (Sigma Stat ver. 3.5, Hulinks, Chicago, IL, USA) and a Bonferroni post-hoc test was employed. *p*-values < 0.05 were considered significant.

## 3. Results

### 3.1. Environmental Conditions

The mean temperature during the Forest condition was significantly lower than that during the Enclosed condition (19.0 ± 0.6* vs.* 17.2 ± 0.7 °C; Enclosed* vs.* Forest, mean ± SEM, *t* = −4.45, *p* < 0.001), whereas the illumination in the Forest condition was significantly higher than that in the Enclosed condition (394 ± 47* vs.* 669 ± 61 lux, *t* = 5.38, *p* < 0.001). Relative humidity showed similar values in the two conditions (35.9 ± 4.5* vs.* 38.4% ± 5.3%, *t* = 0.76, *p* > 0.05).

### 3.2. Physiological Responses

**Figure 2:**
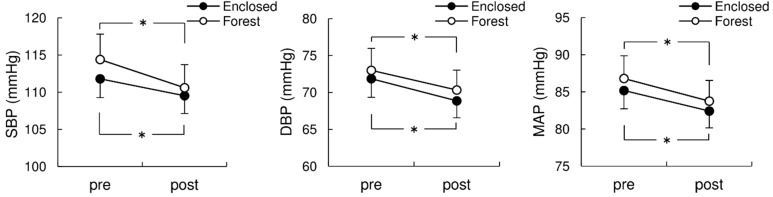
Changes in systolic blood pressure (SBP; left panel), diastolic BP (DBP; middle), and mean arterial pressure (MAP; right) between the Forest and Enclosed conditions. Values are mean ± SEM (*n* = 15). * *p* < 0.05 between pre- and post-values within each condition with the Bonferroni *post-hoc* test.

Table 1 provides the summarized results of the two way repeated measures ANOVA analyses of the subjects’ blood pressure variables. Repeated measures ANOVA revealed significant main effect of the time (pre* vs.* post), while no significant main effect of condition (Enclosed* vs.* Forest) in each BP variables (Table 1). By Bonferroni *post-hoc* test, the pre-experimental stimuli BP variables,* i.e.*, SBP, DBP, and MAP, showed similar values in the two conditions (SBP: Enclosed, 112 ± 3 mmHg, Forest, 114 ± 3 mmHg; DBP: Enclosed, 72 ± 3 mmHg, Forest, 73 ± 3 mmHg; MAP: Enclosed, 85 ± 2 mmHg; Forest, 87 ± 3 mmHg, *p* > 0.05, respectively). After both the Forest and Enclosed conditions, all BP variables were significantly decreased (SBP: Enclosed, 110 ± 2 mmHg, *p* = 0.021; Forest, 111 ± 3 mmHg, *p* < 0.001; DBP: Enclosed, 69 ± 2 mmHg, *p* = 0.015; Forest, 70 ± 3 mmHg, *p* = 0.028; MAP, Enclosed, 82 ± 2 mmHg, *p* = 0.009; Forest, 84 ± 3 mmHg, *p* = 0.005). The interaction between condition and time was not significant (Table 1), indicating that there were no significant differences in BP variations across the two conditions (SBP: Enclosed, −3.9 ± 1.2 mmHg; Forest, −5.3 ± 0.9 mmHg; DBP: Enclosed, −2.9 ± 1.1 mmHg; Forest, −2.7 ± 1.2 mmHg; MAP: Enclosed, −3.2 ± 1.0 mmHg; Forest, −3.6 ± 0.9 mmHg, *p* > 0.05, respectively, Figure 2).

**Table 1:** Results of the two-way repeated measures ANOVA for the blood pressure (BP) variables.

BP	Main Effect						Interaction		
	Condition (Enclosed* vs.* Forest)			Time (pre* vs.* post)			Condition × Time		
	*F*	*p*	η^2^	*F*	*p*	η^2^	F	*p*	η^2^
SBP	2.601	0.125	0.01	17.449 **	0.001	0.02	1.872	0.223	<0.01
DBP	1.537	0.235	0.01	12.143 **	0.004	0.02	0.042	0.841	<0.01
MAP	2.185	0.161	0.01	15.778 **	0.001	0.02	0.047	0.831	<0.01

df = (1,14) was appreciable to each main effect (Condition or Time), and Interaction (Condition × Time). ** *F* is significant at the 0.01 level, and *p* value is the significance probability of each main effect and interaction.

**Figure 3:**
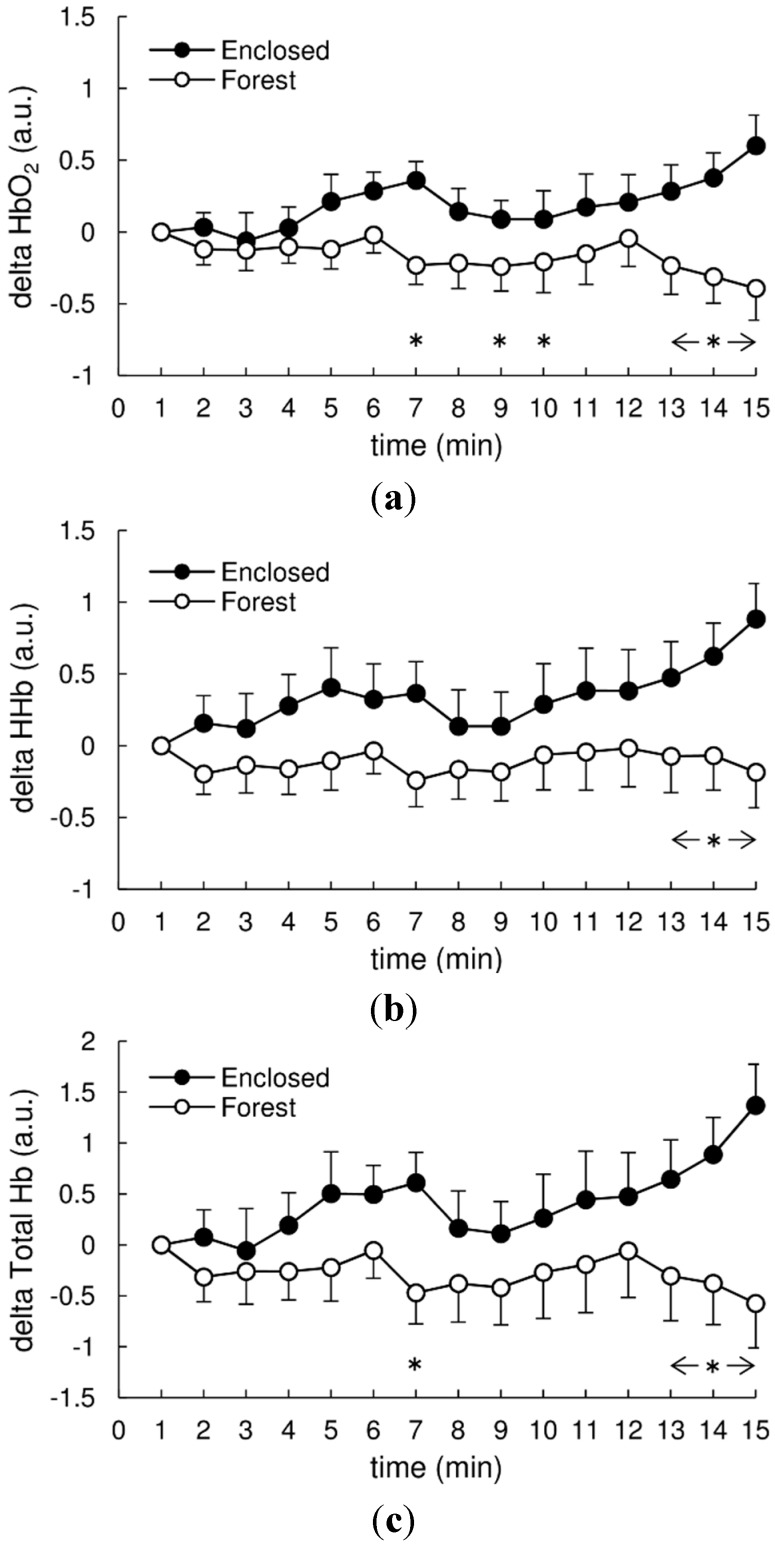
Delta changes in oxygenated hemoglobin (**a**) HbO_2_, deoxygenated hemoglobin (**b**) HHb, and total hemoglobin (**c**) Hb between the Enclosed and Forest conditions. Values are mean ± SEM (*n* = 15). * *p* < 0.05 between the Enclosed and Forest conditions with the Bonferroni *post-hoc* test.

Time course changes in each NIRS signal are shown in Figure 3, and Table 2 provides the summarized results of the two way repeated measures ANOVA analyses of the subjects’ NIRS signals. Repeated measures ANOVA revealed a significant main effect of the condition (Enclosed* vs.* Forest) in HbO_2_, and showed a tendency in HHb and total Hb. In contrast, repeated measures ANOVA revealed a significant main effect of the time (1–15 min) in HHb, while no significant main effect of time was revealed in HbO_2_ and total Hb (Table 2). By Bonferroni *post-hoc* test, each NIRS signal in the Forest condition was significantly lower than that in the Enclosed condition during the later period, in particular, during 13–15 min, of the experimental stimuli (HbO_2_: *p* = 0.002 at 13 min, *p* = 0.002 at 14 min, *p* < 0.001 at 15 min; HHb: *p* = 0.033 at 13 min, *p* = 0.027 at 14 min, *p* = 0.003 at 15 min; total Hb: *p* = 0.012 at 13 min, *p* = 0.010 at 14 min, *p* < 0.001 at 15 min, Figure 3). The interaction between condition and time was also significant in HbO_2_ and total Hb, and also showed a tendency in HHb (Table 2).

**Table 2:** Results of the two-way repeated measures ANOVA of NIRS signals.

NIRS Signals	Main Effect						Interaction		
	Condition (Enclosed* vs.* Forest)			Time (1 min to 15 min)			Condition × Time		
	*F*	*p*	η^2^	*F*	*p*	η^2^	*F*	*p*	η^2^
HbO_2_	7.046 *	0.019	0.12	0.836	0.622	0.01	3.521 ***	<0.001	0.05
HHb	3.710	0.075	0.09	2.052 *	0.019	0.02	1.605	0.087	0.02
total Hb	4.366	0.055	0.10	1.342	0.192	0.02	2.500 **	0.004	0.04

df = (1,14) was appreciable to each main effect (Condition or Time), and Interaction (Condition × Time). * *F* is significant at the 0.05 level, ** *F* is significant at the 0.01 level, and *** *F* is significant at the 0.001 level. *p* value is the significance probability of each main effect and interaction.

With respect to sAMY, the two-way repeated measures ANOVA with the factors of condition (Enclosed* vs.* Forest) and time (pre* vs.* post) revealed no significant main effects of condition, *F*(1,14) = 1.549, *p* = 0.234, η^2 ^= 0.01 or of time, *F*(1,14) = 0.002, *p* = 0.967, η^2^ = 0.00. The interaction between time and condition was also not significant, *F*(1, 14) = 0.586, *p* = 0.457, η^2^ = 0.00 (Table 3).

**Table 3:** Salivary amylase changes in the Enclosed and Forest conditions.

Stress Marker	Condition	Pre			Post		
Salivary amylase (kU/L)	Enclosed	14.3	±	3.3	16.5	±	3.2
	Forest	19.0	±	4.2	16.7	±	3.2

Values are mean ± SEM (*n* = 15).

Table 4 shows the HR variables of both conditions for the first and last 5 min. The results of the two-way repeated measures ANOVA are summarized in Table 5. Repeated measures ANOVA revealed a significant main effect of the time (first 5 min* vs.* last 5 min) in HR and HF, while no significant main effect in LF/HF. Conversely, there was not significant main effect of the condition (Enclosed* vs.* Forest) in HR, HF, and LF/HF (Table 5). The Bonferroni post-hoc test showed significant differences between the first and last 5 min under both conditions (*p* = 0.043, Enclosed condition; *p* = 0.022, Forest condition) in HR. Moreover, the HF was significantly increased during the Forest condition (*p* = 0.009) and showed a tendency to increase in the Enclosed condition (*p* = 0.074). Conversely, no significant changes in LF/HF levels were observed for either condition (*p* > 0.05). The interaction between condition and time was not significant in HR, HF, and LF/HF (Table 5).

**Table 4:** Comparison of heart rate variability (HRV) between the first and the last 5 min during the Enclosed and Forest viewing conditions.

HRV	Condition	0–5 min	10–15 min
HR (bpm)	Enclosed	68.9 ± 2.1	67.6 ± 2.0 *
	Forest	69.0 ± 2.7	67.5 ± 2.3 *
HF (msec^2^)	Enclosed	202 ± 29	231 ± 32
	Forest	204 ± 35	249 ± 26 **
LF/HF	Enclosed	2.12 ± 0.28	1.62 ± 0.46
	Forest	2.04 ± 0.42	1.85 ± 0.67

Values are mean ± SEM (*n* = 15). HF, high frequency (0.15–0.4 Hz); LF, low frequency, 0.04–0.15 Hz. * *p* < 0.05, and ** *p* < 0.01 between the first (0–5 min) and the last 5 min (10–15 min) in each condition with the Bonferroni *post-hoc* test.

**Table 5:** Results of the two-way repeated measures ANOVA of heart rate variability (HRV).

HRV	Main Effect						Interaction		
	Condition (Enclosed* vs.* Forest)			Time (first* vs.* last 5 min)			Condition × Time		
	*F*	*p*	η^2^	*F*	*p*	η^2^	*F*	*p*	η^2^
HR	0.001	0.972	<0.01	6.125 *	0.027	0.01	0.296	0.595	<0.01
HF	0.420	0.528	<0.01	8.074 *	0.014	0.03	0.848	0.374	<0.01
LF/HF	0.066	0.802	<0.01	1.347	0.267	0.01	0.621	0.445	<0.01

df = (1,14) was appreciable to each main effect (Condition or Time), and Interaction (Condition × Time). * *F* is significant at the 0.05 level, and *p* value is the significance probability of each main effect and interaction.

### 3.3. Psychological Responses

Table 6 shows the changes in the subscale scores of the POMS across the Forest and Enclosed conditions, and the results of two-way repeated measures ANOVA are shown in Table 7. Repeated measures ANOVA revealed significant main effect of the time (pre* vs.* post) in all subscale scores of the POMS, while no significant main effect of the condition (Enclosed* vs.* Forest) in all subscales (Table 7). By Bonferroni *post-hoc* test, subscale scores of the Trait-Anxiety (T-A), Depression (D), Fatigue (F), and Confusion (C) in the Forest condition significantly decreased after viewing a real forest, while there were no differences in Anger-Hostility (A-H) and Vigor (V). In contrast, under the Enclosed condition, the scores of only Vigor subscale significantly increased (Table 6). The interaction between condition and time was not significant except the subscale of the Confusion (Table 7).

**Table 6:** POMS scores in the two viewing conditions.

Condition	Enclosed			Forest		
POMS	Pre	Post	*p* value	Pre	Post	*p* value
Trait-Anxiety	3.5 ± 0.7	2.4 ± 0.6	0.234	4.2 ± 0.9	2.2 ± 0.8 *	0.031
Depression	1.2 ± 0.4	0.9 ± 0.4	0.345	1.5 ± 0.5	0.2 ± 0.1 **	0.001
Anger-Hostility	0.5 ± 0.2	0.3 ± 0.2	0.265	0.4 ± 0.2	0.2 ± 0.2	0.265
Vigor	4.3 ± 1.0	2.6 ± 0.7 **	0.008	5.1 ± 0.9	3.9 ± 0.9	0.060
Fatigue	2.6 ± 0.7	1.7 ± 0.5	0.068	2.7 ± 0.6	0.9 ± 0.3 **	0.001
Confuse	5.1 ± 0.6	4.9 ± 0.4	0.795	5.8 ± 0.6	4.1 ± 0.3 **	0.002

Values are mean ± SEM. *p* values mean the difference between pre and post experimental stimuli in each condition. * *p* < 0.05, and ** *p* < 0.01 between the pre and post in each condition with the Bonferroni *post-hoc* test.

**Table 7:** Results of the two-way repeated measures ANOVA of POMS scores.

POMS	Main Effect						Interaction		
	Condition (Enclosed* vs.* Forest)			Time (pre* vs.* post)			Condition × Time		
	*F*	*p*	η^2^	*F*	*p*	η^2^	*F*	*p*	η^2^
T-A	0.185	0.674	<0.01	7.399 *	0.017	0.07	0.483	0.499	0.01
D	0.337	0.571	0.01	17.013 **	0.001	0.07	2.654	0.126	0.03
A-H	0.189	0.670	<0.01	6.000 *	0.028	0.02	<0.001	1.000	<0.01
V	3.684	0.076	0.02	11.663 **	0.004	0.05	0.374	0.551	<0.01
F	0.387	0.544	0.01	14.403 **	0.002	0.10	1.683	0.216	0.01
C	0.019	0.893	<0.01	6.668 *	0.022	0.06	4.996 *	0.042	0.05

df = (1,14) was appreciable to each main effect (Condition or Time), and Interaction (Condition × Time). * *F* is significant at the 0.05 level, and ** *F* is significant at the 0.01 level. *p* value is the significance probability of each main effect and interaction.

## 4. Discussion

The major findings of the present study are that viewing a real forest (the Forest condition) may have more positive effects on cerebral oxygenation and profile of mood states (POMS) subscales than not viewing a real forest (the Enclosed condition), though both conditions showed similar effects on blood pressure, heart rate variability, and salivary amylase changes. These physiological and psychological changes indicate that regardless of the visual stimulus, *i.e.*, with (forest) or without (enclosed) viewing a real forest, forest bathing may have similar positive effects on cardiovascular responses such as blood pressure (BP), heart rate variability (HRV), and saliva amylase (sAMY). In contrast, viewing the real forest heightened cerebral activity, which may be associated with psychological responses compared to without viewing a real forest.

### 4.1. Cardiovascular Responses

Previous studies demonstrated that the blood pressure (BP) levels of individuals in forest environments are significantly lower than those in urban areas [6,7]. Tsunetsugu* et al.* (2007) [7] reported that forest walking reduces diastolic BP (DBP) but not systolic BP (SBP). It was also reported that the decreases in both SBP and DBP following forest walking are significantly greater than the SBP and DBP decreases from walking in urban areas [25]. In contrast, another study demonstrated that both SBP and DBP remain unchanged in both urban and forest environments [8]. These previous studies are partly in agreement with our results. An interesting finding of the present study was that both the Forest and Enclosed conditions had similar effects on BP (*i.e.*, reductions), suggesting that simply being in a forest setting may cause reductions in BP. Indeed, a laboratory study demonstrated that that the smell of wood during cedrol inhalation reduced BP [26,27]. Another possibility is that auditory stimulation may have caused the reductions in BP under both conditions in the present study. Mishima* et al.* [28] reported that BP levels in response to murmur sounds were lower compared to null sound. Although it is assumed that field studies simulate different conditions from laboratory studies with respect to the quality of natural settings and the degree of experimental stimulation, the similar reductions in BP observed in both conditions in the present study may be partly explained by forest immersion *per se* and/or by olfactory and auditory stimulation.

Nevertheless, the mechanism(s) that specifically caused the reductions of BP in both conditions are still unclear. As we calculated BP by multiplying the cardiac output (*i.e.*, stroke volume) by the heart rate (HR) via total vascular resistance, a reduction in BP denotes a potential decrease in stroke volume, HR, and/or vascular resistance. In this study, the subjects’ HR values were slightly but significantly decreased in the last 5 min compared to the first 5 min of the experiment in both conditions, and it is unlikely that the stroke volume had increased due to the complete resting conditions of the study. Thus, reductions in BP variables may be caused by autonomic nervous system activity.

We evaluated the autonomic nervous system using heart rate variability (HRV), and we compared its state in the first 5 min of the 15-min viewing with its state in the last 5 min of viewing. The period of 5 min, which was used for the further analyses, is a well-established measure used to chart stable physiological responses [16]. Previous studies found that viewing nature increases parasympathetic nerve activity assessed through HRV [29,30]. In agreement with these studies [29,30], our results also showed that high-frequency oscillations (HF), as an indicator of parasympathetic nerve activity, were significantly increased in the Forest condition and tended to increase in the Enclosed condition (*p* = 0.074). Although the LF/HF ratio as an indicator of sympathetic nerve activity did not show differences between the first and last 5 min in either condition, it is reasonable that increased parasympathetic nerve activity may be associated with the decrease in BP variables. Taken together, the results of BP variables and HRV were similar in two conditions, and these results indicate that the main effect of the time (pre* vs.* post) strongly affected these responses rather than the effect of condition (Enclosed* vs.* Forest) as no significance was observed in the main effect of the condition and interaction (condition × time). 

Previous studies have shown that sympathetic nerve activity assessed through direct measurements of sympathetic nerve activity using microneurography,* i.e.*, muscle sympathetic nerve activity, may help regulate BP [31]. In addition, the indirect assessment of sympathetic nerve activity through HRV was associated with BP control in pre-hypertensive humans [32]. It was also reported that HRV can be a prognostic indicator of cardiovascular disease [33]. Thus, although the precise mechanisms and interactions between changes in BP and HRV are still unclear, the present findings indicated that viewing the forest did not have more benefit in regard to cardiovascular responses compared to not viewing the forest in the same setting, indicating that being present in a forest setting without viewing the forest may have potential advantages in environmental planning. In other words, our findings indicate that being in a forest setting may be of benefit even without time spent viewing the forest. Therefore, this information may be useful for urban environmental planning even under the condition without viewing a real forest.

### 4.2. Cerebral Oxygenation

In this study, the subjects’ oxygenated hemoglobin (HbO_2_) levels in the Forest condition were decreased in the latter phase. Similarly, total Hb as an indicator of blood volume showed a similar trend under the Forest condition. Park* et al.* (2007) [5] reported that total hemoglobin (Hb) decreased significantly more following forest viewing compared to urban viewing, suggesting that *shinrin-yoku* has a relaxing effect. Although the mechanisms that caused the present subjects’ HbO_2_ to decrease under the Forest condition are unclear, it is likely that viewing a real forest had a positive effect on the subjects’ cerebral activity. A possible explanation could be that comfortable feelings may affect cerebral oxygenation. Geroge* et al.* [21] demonstrated that pleasant feelings cause a significant decrease in cerebral blood flow (CBF). Similarly, screen image studies have shown that while pleasant feelings decrease HbO_2_, unpleasant feelings increase it [11]. In addition, Hoshi* et al.* [20] manipulated the CBF while measuring NIRS signals in an animal model at rest and demonstrated that HbO_2_ is sensitive to changes in CBF; e.g., HbO_2_ is augmented by increases in CBF. Thus, decreases in HbO_2_, which were observed under the present Forest condition, might reflect decreases in CBF caused by feelings of relaxation.

Surprisingly, both HbO_2_ and deoxygenated hemoglobin (HHb) under the Enclosed condition continued to increase until the end of the 15-min viewing and showed significantly different trends across the final 5 min phases of the Forest and Enclosed conditions. NIRS has been widely used for the assessment of cerebral neural activation, as it reflects changes in cerebral oxygenation [34]. However, several previous studies reported HHb increases and HbO_2_ decreases during cerebral neural activation in patients with restricted CBF, such as those suffering from cerebral ischemia [35,36] or Alzheimer’s disease [37], those of advanced age [36,38], and those experiencing exercise-induced changes [39]. These results are inconsistent with our present findings. The mechanism that caused the increase in HbO_2_ in the Enclosed condition despite the increase in HHb remains unclear. This may be explained by the previous finding of more increase and enough regional CBF, which is required for O_2_ consumption in the brain [40]. One may also speculate the possibility of increased neuronal activity, indicating an increase in HHb despite an increase in HbO_2_. However, interpreting this trend is complicated and further research is needed.

### 4.3. Salivary Amylase

In the present study, we evaluated the salivary amylase (sAMY) level as a stress marker because saliva sampling is a noninvasive method, making the sample collection straightforward and stress-free for the subjects. It has been reported that increased sympathetic nerve activity is a major stimulator of amylase secretion, indicating that salivary amylase activity can be a useful index for plasma noradrenaline levels under various stressful conditions [9,22]. A previous study demonstrated that sAMY levels in individuals exposed to a forest environment were lower than those in individuals in urban environments, where both sets of individuals had walked in and observed the surrounding landscape [9]. Similarly, a recent field study also revealed that sAMY under a very natural environment was significantly lower compared to a built setting [41]. In addition, similar stress markers evaluated using saliva, e.g., salivary cortisol concentrations, were significantly lower for subjects in forest environments compared to those in urban areas after viewing the surrounding landscape [5,6,7,8], and it was reported that greater green space was associated with lower salivary cortisol concentrations [42].

Although our study did not compare forest and urban environments and no difference in sAMY was observed for either the Enclosed or Forest condition, our results seem to be inconsistent with previous studies. However, it should be noted that these previous studies compared sAMY or cortisol between urban and forest environments within the same time period (e.g., within pre-stimuli and within post-stimuli) between urban and forest conditions, and it was reported that a significant difference was observed between urban and forest conditions within same time period [5,6,7,8]. Thus, these studies did not investigate whether the forest environment *per se* reduced salivary stress markers, indicating that our results, which showed no difference between pre- and post-experimental stimuli or between the Enclosed and Forest conditions, may not be contrived [5,6,7,8]. 

Another explanation may involve the effect of diurnal rhythms. In fact, a recent study suggested that the measurement of saliva samples should consider the effects of diurnal rhythms [43]. In the present study, each participant underwent the two conditions with a 30-min interval; our experimental schedule was arranged to eliminate circadian rhythms. However, these contributing factors may have affected the sAMY values of our subjects.

### 4.4. Profile of Mood States (POMS)

Several laboratory studies have demonstrated that viewing “nature” may have a positive effect on psychological outcomes [30,44]. Similarly, it was reported that green space improves the self-esteem and mood of clinical populations [1]. Field studies conducted in forests have investigated the effects of forest environments on POMS scores [6,8] and on other factors [4,5,7]. It was reported that all POMS subscales were improved through forest viewing and walking [6,8]. Similar results including improvements in feelings of hostility, depression, boredom, friendliness, well-being, comfort, calm, liveliness, and refreshment have also been observed [4,5,7].

Although these results appear to be consistent with our findings in that the forest environment improved the subjects’ POMS subscale scores, it is interesting that the two-way ANOVA revealed significant improvements only in the Forest condition, although the Enclosed condition improved POMS scores without significant differences. These results indicated that forest setting *per se*,* i.e.*, either viewing or not viewing a real forest, may have a beneficial effect on POMS. Otherwise, POMS was strongly affected by the main effect of time (pre* vs.* post) rather than the main effect of condition (Enclosed* vs.* Forest) as no significant main effect of the condition and interaction (time × condition). To our knowledge, no prior study has examined POMS changes across conditions including and omitting forest viewing. It may therefore be difficult to explain these differences in POMS scales across the Enclosed and Forest conditions. However, it is intriguing to consider an interaction between cerebral activity and psychological factors. Animal studies have demonstrated that the dorsolateral prefrontal cortex is an important area that provides information from the amygdala, which controls emotion [45,46]. In addition, a recent study suggested that the dorsolateral prefrontal cortex may play an important role in regulating feelings in humans [47]. The results of the present study showed similar tendencies in HbO_2_ signals and POMS across the Enclosed and Forest conditions. For example, HbO_2_ decreased under the Forest condition but increased under the Enclosed condition. Moreover, although the POMS subscales under the Forest condition improved, no significant changes were observed under the Enclosed condition. As noted above, previous laboratory studies have demonstrated that comfortable feelings are associated with a reduction in HbO_2_ [11].

Taken together, the present finding of significant improvements in POMS scores might be associated with increased feelings of comfort as assessed through NIRS signals, although whether it is the NIRS signals or the POMS improvement that comes first is unknown. Our results indicate that visual stimulation might be required for and may accentuate the psychological benefits to human health compared to not viewing a real forest, unlike the case of physiological effects.

### 4.5. Technical Considerations

Several limitations must be considered when interpreting the findings of the present study. First, the environmental conditions,* i.e.*, ambient temperatures and illumination levels were significantly different across the two conditions. In general, lower ambient temperatures have been shown to reduce the subjects’ HR [48] and increase their SBP and DBP [49]. However, previous studies have been conducted under even greater changes in ambient temperature,* i.e.*, 21 °C* vs.* 28 °C [48] and 15 °C* vs.* 0.5 °C [49]. Moreover, in the aforementioned study, the 7 °C change from 15 °C–8 °C did not alter the subjects’ SBP and DBP values [49]. In our study, although significant differences in temperature were observed, the differences were much smaller than those of the previous study [49]. Moreover, the baseline values of the subjects’ BP variables were similar, indicating that the effect of different ambient temperatures was probably minimal in this study. In addition, we cannot clarify and rule out the effect of different illumination levels on physiological and psychological responses in two conditions completely. However, ambient illumination would be one factor of the landscape as well as enclosed or forest conditions. Moreover, it is known that the pupil can well regulate in response to the intensity (luminance) of light to adapt to various levels of lightness/darkness [50]. Thus, we do believe that the illumination does not cause much of a difference to the subjects’ physiological and psychological responses, although further research is needed. 

Second, we could not completely rule out the effect of skin blood flow on the left frontal lobe. We recently reported that during dynamic exercise, HbO_2_ can be influenced by thermoregulatory changes in skin blood flow, and therefore may not be completely reflective of cerebral oxygenation [51]. However, in the present study, the subjects maintained a complete resting condition while sitting in a comfortable chair, and the environmental temperature remained almost unchanged throughout the experiment. Moreover, based on the calculations derived from the NIRS technique, our device minimized the effect of skin blood flow [18,19]. Therefore, it is unlikely that changes in the skin blood flow in the forehead affected the NIRS signals in the present study. 

Finally, it is known that HRV may be affected by respiration rhythm [52,53], and the appropriate control of respiration is therefore necessary in assessing sympathetic and parasympathetic nerve activities using HRV. In contrast, modifications of respiratory rhythm between 0.12 and 0.35 Hz did not affect the peak frequency of HR in both the supine and standing positions [54]. In the present study, we did not use a metronome to control each individual’s respiration rhythm, and the subjects were only asked to maintain a constant respiration rhythm and avoid hyperventilation and breath-holding, because the study aimed to measure the influence of forest viewing, and we did not want to introduce other stimuli such as a metronome sound. Consequently, further studies that assess sympathetic nerve activity using HRV are warranted.

## 5. Conclusions

In summary, our results suggest that viewing a real forest may be associated with feelings of comfort which result in a decrease in HbO_2_ and a reduction in psychological stress. Our findings suggest that visual stimulation might be required for and accentuate psychological benefits in human health compared to not viewing a real forest, while similar effects on BP and HR variables may occur either with (Forest condition) or without (Enclosed condition) viewing a real forest. This information is important to understanding how natural environments—e.g., viewing or not viewing a real forest, but exposure in the same forest setting—contribute to human health. Collectively, although sitting in a forest setting with or without viewing the forest influenced mostly psychological benefits, our findings suggest that potential physiological benefits can be expected even in an environment without the visual perception of a forest. The present results may therefore provide helpful information to expand the range of choices for the design and planning of “forest bathing” environments, which can be applied under artificial and/or urban environments for human health.
